# Genetics of animal health and disease in cattle

**DOI:** 10.1186/2046-0481-64-5

**Published:** 2011-03-31

**Authors:** Donagh P Berry, Mairead L Bermingham, Margaret Good, Simon J More

**Affiliations:** 1 Animal and Grassland Research and Innovation Centre, Teagasc, Moorepark, Co. Cork, Ireland; 2 The Roslin Institute and Royal (Dick) School of Veterinary Studies, University of Edinburgh, Roslin, Midlothian EH25 9PS, UK; 3 Department of Agriculture, Fisheries and Food, Kildare St., Dublin 2, Ireland; 4 Centre for Veterinary Epidemiology and Risk Analysis, UCD School of Agriculture, Food Science and Veterinary Medicine, University College Dublin, Belfield, Dublin 4, Ireland

## Abstract

There have been considerable recent advancements in animal breeding and genetics relevant to disease control in cattle, which can now be utilised as part of an overall programme for improved cattle health. This review summarises the contribution of genetic makeup to differences in resistance to many diseases affecting cattle. Significant genetic variation in susceptibility to disease does exist among cattle suggesting that genetic selection for improved resistance to disease will be fruitful. Deficiencies in accurately recorded data on individual animal susceptibility to disease are, however, currently hindering the inclusion of health and disease resistance traits in national breeding goals. Developments in 'omics' technologies, such as genomic selection, may help overcome some of the limitations of traditional breeding programmes and will be especially beneficial in breeding for lowly heritable disease traits that only manifest themselves following exposure to pathogens or environmental stressors in adulthood. However, access to large databases of phenotypes on health and disease will still be necessary. This review clearly shows that genetics make a significant contribution to the overall health and resistance to disease in cattle. Therefore, breeding programmes for improved animal health and disease resistance should be seen as an integral part of any overall national disease control strategy.

## Background

There continues to be very significant advances in efforts to control disease in cattle, with the potential for significant improvements to both performance and welfare. These advances have included improved understanding of disease pathophysiology and epidemiology, as well as the development of products such as antibiotics and anthelmintics for improved disease control.

Concurrently, there have been considerable advances in animal breeding and genetics, relevant to animal disease control. These advances are of considerable veterinary interest, noting that observed animal performance is the outcome of the interaction between the animal's genetic makeup and the specific environment it was exposed to. Logically, therefore, improved genetics has the potential to complement current approaches to animal disease control. Improvement in animal health through genetic selection is advantageous, because genetic gain is cumulative and permanent, as the genes introduced into a population can persist for many generations. Unravelling the genetic architecture of health and disease resistance not only facilitates knowledge development on potential for breeding for improved health status but also generates knowledge for biomedical research in animals and humans including applications such as vaccine development.

The objective of this review is to summarise research studies on the genetics of animal health and disease resistance in cattle, with particular reference to studies undertaken in Irish cattle. The implications of these results in breeding for improved animal health and disease resistance are discussed.

## Genetic terminology

Prior to discussing the genetics of animal health and disease resistance, the terms commonly used by animal breeders to describe the characteristics of a population need to be explained:

### Phenotype

The phenotype is simply the observed performance of an animal "in the field" (e.g., dystocia in cows or the presence or absence of infection as measured by a positive or negative diagnostic test result). A phenotype, or trait, may be continuous (also called quantitative; e.g., milk yield and growth rate) or discrete (qualitative; e.g., did or did not succumb to disease).

### Genotype

The definition of genotype varies. Animal breeders commonly use genotype to describe a particular strain of animal (e.g., animals of a given breed from a particular origin). Molecular geneticists, however, commonly use genotype to describe the genetic variants (i.e., alleles) an individual possesses at a particular position in its DNA, also known as a locus.

### Genetic markers

A genetic marker is a measurable variation in the DNA sequence of a population. Common types of genetic markers, generally termed polymorphisms, include microsatellites, indels (i.e., insertions or deletions of pieces of DNA), single nucleotide polymorphisms (SNPs - pronounced *"snips"*) and copy number variants (CNVs).

### Quantitative trait locus (QTL)

A segment of a chromosome that has been experimentally demonstrated to be statistically associated with variation in a quantitative or complex phenotypic trait.

### Heritability

Heritability summarises the proportion of phenotypic variation, or differences among a cohort of animals, attributable to genetic variation between individuals. Animal breeders commonly cite the narrow-sense heritability, denoted as *h*^2^, which is the proportion of phenotypic variation attribute to additive genetic variation (i.e., genes passed on from one generation to the next). In the calculation of broad-sense heritability, the numerator also includes non-additive genetic variation. In this review we will only consider narrow-sense heritability estimates. Heritability varies from 0 (not heritable) to 1 (fully heritable). If the heritability is high, we can expect a large proportion of the phenotypic differences of the parents to be passed on to the progeny. Also, the larger the heritability, the greater the expressed phenotype resembles the genetic merit of the animal.

### Maternal heritability

Maternal heritability is the proportion of phenotypic variation among offspring that is due to the genes expressed by the dam. A maternal heritability of calving difficulty is often quoted which encompasses for example, the size of the pelvis of the dam. The direct heritability for calving difficulty is the effect of the genes of the dam (and sire) on, for example, the size of the calf. A maternal heritability has also been reported for weaning weight [1] which includes genetic characteristics of the dam such as her milk yield.

### Genetic variation

A measure of the variation or differences within a population that is due to the differences in genetic merit of the animals. More commonly, genetic variation is expressed as the genetic standard deviation (i.e., the square root of the genetic variance) within a population.

### Genetic correlation

A genetic correlation describes the strength of the linear relationship between two traits due to the genetic influences on each trait. It varies from -1 (strong negative relationship) to 0 (no relationship) to +1 (strong positive relationship between two variables). Genetic correlations can be due to the same mutation affecting both traits (termed pleiotropic effect) or different mutations affecting both traits but tending to, on average, be inherited together (i.e., linked).

### Estimated breeding value (EBV)

Estimated breeding value is an estimate of the genetic merit for an animal for a given trait or series of traits based on an evaluation of all available data on the performance of an animal, and close relatives, for a trait. Using traditional methods of genetic evaluation, the true breeding value (or true genetic merit) is not known. The estimates of genetic merit are generally presented as the predicted transmitting ability (PTA) in dairy cattle or expected progeny difference (EPD) in beef cattle which are the EBV divided by two (i.e., an animal only passes half its genes to its progeny).

## Basis of genetic gain

Annual genetic gain for a given trait may be described by the following equation [2]:

where ΔG is annual genetic gain; i is the intensity of selection; r is the accuracy with which the genetic merit of each animal is known, σ is the genetic standard deviation (i.e., the square root of the genetic variance or simply just a measure of the genetic differences among animals); and L is the generation interval.

**Figure 1:**
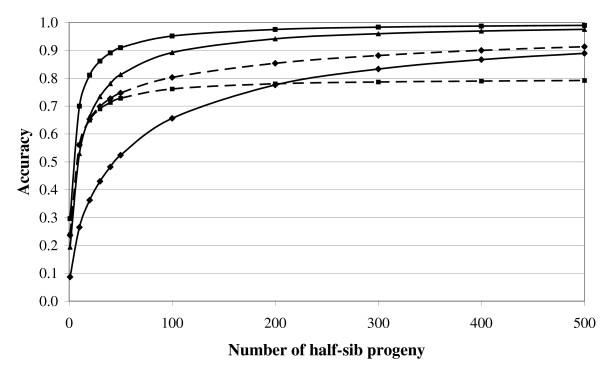
**Accuracy of selection across different number of half-sib progeny based on direct selection where the heritability of the trait is 0.03 (diamond with continuous line), 0.15 (triangle and continuous line), 0.35 (square with continuous line) and indirect selection where the goal trait is the 0.03 heritability trait and data is available on the 0.35 heritability trait alone (square with broken line) or also available on the 0.03 heritability trait (diamond with broken line), assuming a genetic correlation of 0.80 between both traits**.

The greater the selection intensity the greater will be the genetic gain for that trait. The accuracy of selection is affected by both the heritability of the trait and the information available on the animal itself and its relatives. Figure 1 illustrates how the accuracy of selection, ignoring pedigree contributions, increases as the number of half-sib progeny with records increases across different heritability estimates. For a given number of progeny, the accuracy will be greater for higher heritability traits. However, accuracies of near unity are still achievable even for low heritability traits if sufficient information is available. Therefore, with the appropriate breeding programme (i.e., large paternal half-sib groups) and infrastructure for the collection and storage of data, genetic gain in low heritability traits is certainly achievable if genetic variation is present. The accuracy of selection for a given trait may also be augmented by indirect selection for a correlated trait (Figure 1). The genetic standard deviation is a measure of the variation present in the population and the generation interval is the average age of the parents when its progeny are born. This is approximately 6 years in Irish dairy and beef cattle [3], which is consistent with international estimates.

## Health and disease in cattle

### Viral diseases

There are many bovine viral diseases in Ireland, including bovine viral diarrhoea (BVD) and several viral infections associated with the respiratory system (infectious bovine rhinotracheitis [IBR], bovine parainfluenza-3 [PI-3] and bovine viral syncytial virus [BRSV]). As yet, there has been no Irish research to quantify the genetic variation present in susceptibility to bovine viral diseases.

Breed differences in susceptibility to respiratory disease, however, have been demonstrated elsewhere [4]. Furthermore, heritability estimates different from zero have also been reported, highlighting the presence of within-breed genetic differences in susceptibility to respiratory disease. Heringstad et al. [5] estimated a heritability of 0.05 (SE = 0.018) for respiratory disease in Norwegian calves, with a genetic standard deviation of 2 percentage units. Norway is free from IBR and BVD so the most prevalent agents in respiratory disease in Norway are BRSV and PI-3.

As part of a larger scale study with 12 different breed types in the USA, Snowder et al. [4] reported a heritability of susceptibility to respiratory disease in pre-weaned calves of between 0.00 and 0.26 (SE = 0.04); the standard deviation varied from zero to 1.47 percentage units. Furthermore, a significant maternal heritability of between 0.00 and 0.13 (SE = 0.07) was observed. This significant maternal heritability indicates that, independent of the genes transmitted to the offspring from the dam, the genetic makeup of the dam herself, either through her uterine environment or post-natal influence (e.g., immunoglobulins present in colostrum), also influences the genetic susceptibility of the calf to respiratory disease. In a follow-up study, Snowder et al. [6] estimated the heritability for respiratory disease in weaned feedlot calves (average age of 176 days) of between 0.04 (SE = 0.01) and 0.08 (SE = 0.01), while the genetic standard deviation varied from 0.57 to 1.17 percentage units. In an independent US study, Schneider et al. [7] reported a heritability of resistance to respiratory disease in pre-weaning and post-weaning calves of 0.11 (SE = 0.06) and 0.07 (SE = 0.04), respectively. These estimates all suggest that there is indeed genetic variation in susceptibility to viral respiratory disease.

### Udder health

Mastitis is one of the most costly diseases in dairy cattle production systems and is also likely to impact significantly on profitability in suckler beef production systems. A plethora of international scientific studies have been undertaken on the genetics of udder health, including genetic analysis of Irish Holstein-Friesian dairy cows [8]. Heritability estimates for mastitis have been variable across studies, reflecting variation in a multitude of factors such as the person undertaking the recording (i.e., farmer or veterinarian) and how they interpret the clinical signs, the completeness of data recording (i.e., some observations not recorded), as well as the pathogen and the environment, including exposure, which may influence the expression of an animal's genotype. Based on a detailed review of literature present at the time, Mrode and Swanson [9] reported a weighted average heritability of lactation average somatic cell count (SCC) and mastitis in primiparae of 0.11 (SE = 0.04) and 0.04, respectively. A recent heritability estimate for mastitis of 0.05 (SE = 0.005) in Irish Holstein-Friesian dairy cows [8] is consistent with this review. The genetic standard deviation for mastitis varies from 1.2 to 7.0 percentage units, suggesting that genetic gain can be achieved by selecting for clinical mastitis. Using data from long-term controlled experiments in Norway, Heringstad et al. [10] showed that a reduction in genetic merit to susceptibility to clinical mastitis is achievable with active selection of sires of superior genetic merit for clinical mastitis. After five generations of selection, cows actively selected for low clinical mastitis had a mean incidence of less than 5% while the incidence of clinical mastitis in cows on the selection lines for increased production was over 25% [10]. A concurrent decline in incidence of ketosis, but not retained foetal membranes (RFM), was also observed [10].

In their review, Mrode and Swanson [9] reported the mean genetic correlation between SCC and mastitis to be ~0.70; this indicates that SCC is a good genetic predictor of mastitis and can, therefore, be used in breeding programmes to augment the accuracy of selection, even where data on clinical mastitis may be available (Figure 1). Recent estimates from Ireland are consistent with these estimates [8]. Nonetheless, despite the relatively strong genetic correlation between SCC and mastitis, indicating that they are both measuring similar genetic characteristics of udder health, the correlation is not unity and, therefore, additional genetic gain can be achieved by recording clinical mastitis, even if data on SCC are available, and used in genetic evaluations.

### Lameness

After mastitis, lameness is probably the next most costly disease in dairy and beef production systems. Heritability of lameness varies from 0.03 to 0.096 when scored by farmers [8,11,12] or by trained assessors [13]. Using data on lameness in Canadian Holstein cows scored by either farmers or veterinarians, Van Dorp et al. [14] reported a heritability for lameness of 0.16; the genetic standard deviation calculated from the provided estimates of the incidence and heritability was 9.5 percentage units. The heritability of lameness in Irish Holstein-Friesian dairy cattle based on predominantly farmer-recorded data was 0.04 (SE = 0.005) [8]. Heritability estimates for different claw disorders (interdigital dermatitis, heel erosion, sole haemorrhage, digital dermatitis, white line disease, interdigital hyperplasia, sole ulcer and chronic laminitis) varied from 0.01 (SE = 0.01) to 0.10 (SE = 0.02) in Dutch multiparous Holsteins [15]. The lowest heritability estimates were for sole ulcers and chronic laminitis, while the highest estimates were for digital dermatitis and interdigital hyperplasia.

### Other bacterial diseases

A broad range of other diseases in Irish cattle can be attributed to bacterial infection. Of these, bovine tuberculosis (TB; caused by infection with *Mycobacterium bovis*) is perhaps the most problematic. There have been intensive national efforts in Ireland towards *M. bovis *eradication for many years [16].

Several studies have attempted to estimate genetic parameters for bacterial diseases. The heritability of susceptibility to *M. bovis *infection in cattle is described in two recent studies [17,18], including one study in Irish Holstein-Friesian dairy cows [17]. Heritability estimates on the observed and liability scales varied from 0.06 to 0.18; standard errors varied from 0.012 to 0.044. The genetic standard deviation for the traits varied from 17 to 23 percentage units. Heritability estimates were based on response to the tuberculin test, and on the presence of TB lesions, as confirmed during abattoir inspection. These results clearly show that significant improvement can be made through genetic section towards TB resistance.

Few genetic parameters are available on the susceptibility to *Mycobacterium avium *subspecies *paratuberculosis *(MAP), the causative organism for Johne's disease, and these have generally been confined to analysis of antibody response to MAP. Heritability estimates vary from 0.05 to 0.15 [19-22], with estimates of between 0.07 to 0.15 (SE varying from 0.024 to 0.062) for Irish Holstein-Friesian dairy cows [23]. The genetic standard deviation for serological response to MAP was calculated from the results of Berry et al. [23] as 5.4 percentage units.

As yet, no attempt has been made to estimate the heritability for susceptibility to salmonellosis in cattle. However, Wray and Sojka [24] reported that Friesian calves are more resistance to *Salmonella typhimurium *infection than Jersey calves, which suggests that genetic differences do exist. Furthermore, heritability estimates for mortality, survival time and quantitative caecal salmonella carrier state at the end of the rearing period for experimentally infected chickens was 0.12, 0.06 and 0.09, respectively [25], which suggests that some genetic variation may also exist in other species.

Like salmonellosis, no estimate of the heritability of brucellosis is available, although Templeton et al. [26] increased natural resistance to brucellosis in calves from 20% to 59% following the breeding of dams to a naturally resistant bull; this suggests that genetic variation is indeed present.

### Metabolic diseases

Some research has been undertaken to investigate the heritability of metabolic diseases of cattle, including ketosis, hypocalcaemia (i.e., milk fever), hypomagnesaemia (i.e., grass tetany) and displaced abomasum. In general, heritability estimates for these metabolic diseases vary from 0.009 to 0.31. Heritability estimates for hypocalcaemia vary from 0.01 to 0.13 [11,27-30]; the standard deviation varied from 0.3 to 4.0 percentage units. The heritability for ketosis varies from 0.01 to 0.16 [28-31]; the standard deviations vary from 0.9 to 1.2 percentage units. There appears to be only one heritability estimate for hypomagnesaemia in dairy cattle of 0.004 (SE = 0.004) [29]. However, heritability estimates for other health traits in that study [29], based on farmer-recorded data, were, in general, lower than estimates from other studies. Heritability estimates for displaced abomasum varies from 0.15 to 0.31 [28,31]. There appears to be no estimate of heritability for acidosis in cattle.

The larger heritability for displaced abomasum relative to the other diseases may be surprising given the known influences of nutrition and management on each of these metabolic disorders. However, the larger heritability may not in fact be due to a truly greater influence of additive genetics on the trait but due to a more accurate recording of the trait since most will be diagnosed by a veterinarian. Furthermore, displaced abomasum is a binary trait, with no ambiguity with respect to the severity of disease.

### Fertility-related conditions

Most diseases impact in some way on reproductive performance. For the purpose of this review, fertility-related diseases are confined to RFM, metritis and cystic ovaries. In a study with over half a million Norwegian Red first lactation cows [31], the heritability of cystic ovaries, metritis and RFM were 0.07 (SE = 0.01), 0.03 (SE = 0.01) and 0.06 (SE = 0.01), respectively. The heritability estimates reported by Heringstad [31] were similar to those reported in other studies for cystic ovaries of between 0.02 and 0.22 [12,14,28,32], for metritis of between 0.04 and 0.06 [12,14,33], and for RFM of between 0.004 to 0.08 [14,34,35]. Furthermore, the genetic correlation between RFM expressed in first, second and third lactation were all greater than 0.54 (SEs were less than 0.062) suggesting that the same genes may be influencing this disorder irrespective of parity [30]. No correlations for either metritis or cystic ovaries across lactation are available.

Heringstad [31] documented a genetic correlation between metritis and RFM of 0.64 (SE = 0.10), indicating that selection for one will also reduce the incidence of the other. In contrast, Van Drop et al. [14] reported a near zero genetic correlation (0.06) between the two traits; no standard error was provided but the estimate was based on 12,471 lactations compared to over half a million by Heringstad [31]. Heringstad [31] failed to identify a correlation between metritis and cystic ovaries (0.14; SE = 0.15) agreeing with Van Dorp et al. [14] who reported a correlation of 0.03. Although Pösö and Mäntysaari [33] reported a moderate to strong positive correlation between metritis and cystic ovaries (0.59), the associated standard error was large (0.33) suggesting it was not different from zero. The genetic correlation between cystic ovaries and RFM was close to zero [14], although Heringstad [31] reported a negative correlation of -0.26 (SE = 0.13) between the traits. Large standard errors were associated with the correlations, due primarily to the low heritability of the respective traits, thereby increasing the uncertainty of the results. However, the lack of any strong genetic correlation between the disease conditions suggests that they are under different genetic control and need to be selected for individually.

### Endo- and ecto-parasites

Although much of the research on the genetics of susceptibility to both endo- and ecto-parasites has been undertaken in sheep [36], there is evidence suggesting genetic variation exists among cattle in susceptibility to parasites. Pan et al. [37] reported a heritability of 0.084 to 0.124 (SE = 0.025 to 0.042) for susceptibility to *Neospora caninum *in Canadian Holstein cattle. Heritability estimates for gastrointestinal nematode burden, measured as eggs per gram of faeces or larvae per gram of faeces, ranged from 0.00 to 0.25 (SE = 0.02 to 0.05) in Dutch Holstein-Friesian cattle [38]; the average was 0.09. Davis [39] in a review of cattle studies reported an average heritability of resistance to ticks of 0.31, a heritability for resistance to buffalo fly of 0.21, and a heritability, excluding the reported estimate greater than one (heritability cannot be greater than one), of 0.32 for faecal egg count.

## Impact of previous and current breeding goals on animal health and susceptibility to disease

It is not possible to accurately quantify genetic trends for most health traits in most countries because accurate records and data on animal health are not available, certainly not routinely. Nonetheless, past genetic trends may be predicted based on estimated genetic correlations with production traits, and the impact of these correlations quantified using knowledge on past breeding goals.

With several notable exceptions, particularly from the Nordic countries, breeding programmes of the past selected aggressively for increased milk production [40]. Current international breeding goals now generally include functional traits such as fertility, udder health, type traits and functional survival [40]. Nonetheless, a large proportion of the relative emphasis in international breeding goals is still on milk production and, therefore, genetic trends can be approximated based on genetic correlations with milk production.

Few estimates are currently available on the genetic correlation between milk production and several infectious diseases. Only one study has reported a genetic correlation between 305-day milk yield and serological response to MAP [23]. Dependent on the parity of animal and dataset used, the genetic correlation varied from -0.44 to 0.07 (standard errors varied from 0.083 to 0.228) in that population of Irish Holstein-Friesian dairy cows. A genetic correlation of -0.48 (SE = 0.13) was observed between 305-day milk yield and bovine tuberculosis in multiparous UK Holstein-Friesian dairy cattle [18], while the genetic correlations with milk, fat and protein yield in Irish Holstein-Friesian cattle varied from -0.31 to 0.39 (standard errors varied from 0.13 to 0.20) [41]. This suggests that selection for increased milk production alone may increase genetic susceptibility to MAP and possibly also bovine TB.

Table 1 summarises the genetic correlation, across a range of international studies from different breeds of dairy cattle, between milk production and several diseases. Although there were a few exceptions, selection for increased milk production alone without any cognisance of other traits is expected to increase the incidence of mastitis, lameness, cystic ovaries, ketosis and metritis. The impact on RFM is less clear.

**Table 1:** Genetic correlations between various health traits and 305-day milk yield in dairy cattle

**SCC**^**1**^	Mastitis	Lameness	Milk Fever	Cystic ovaries	Metritis	Ketosis	**RFM**^**2**^	**DA**^**3**^	Country	Reference
0.06 to 0.82									Multiple	[9]^4^
-0.97 to 0.48									Multiple	[9]^5^
	0.21 (0.06)	0.29 (0.11)	0.19 (0.06)						UK	[11]
	0.09	0.02		0.10	-0.05	0.12			US	[12]^6^
	0.15	0.24		0.23	0.02		-0.28		Canada	[14]
			0.27 (0.17)				0.26 (0.23)		US	[27]
	0.37			-0.14					Canada	[28]
		0.27^7^	-0.67	-0.06		0.77		-0.04	Canada	[28]
	0.35 (0.10)		0.27 (0.12)	0.70 (0.15)					UK	[29]
				0.34 (0.11)					Netherlands	[32]
				0.42 (0.08)	0.68 (0.23)				Finland	[33]
0.10 (0.08)	0.46 (0.09)								Finland	[56]
-0.11 to 0.00 (0.10 to 0.11)	0.35 to 0.61 (0.10 to 0.11)								Finland	[56]
0.15 (0.06)	0.45 (0.09)								France	[57]
0.198 (0.110)		0.056 to 0.34 (0.12 to 0.15)^7^							Germany	[58]
	0.51					0.65			Norway	[59]
	0.29 (0.08)								Germany	[60]
			-0.49						Norway	[61]

^1^Somatic cell count; ^2^Retained foetal membranes; ^2^Displaced abomasum; ^3^Based on nadir calcium levels around parturition; ^4^Based on a review of 7 scientific papers; ^5^Based on a review of 9 scientific papers; ^6^Estimates based on product moment correlations between estimated breeding values^; 7^Lameness based on claw and hoof disorders or culling for leg problems;

## Limitations of the current methodology in animal breeding

Traditional genetic evaluations use sophisticated statistical methodology to estimate the genetic merit of an animal, and require large datasets containing information on animals, their pedigree, sibs and progeny. These methods of genetic evaluation are not without their limitations, particularly when seeking to breed for improved resistance to disease. Examples of the limitations include:

• the phenotype measured contains error (i.e., variable diagnostic accuracy depending on the farmer or the veterinarian),

• the phenotype may not be measurable in both genders (e.g., mastitis in cows),

• performance in adult cattle cannot be measured in calves, thereby delaying the time lag to identification of genetically different animals for adult performance,

• antagonistic or unfavourable genetic correlations between traits of interest cannot be easily resolved (e.g., health and milk production), and

• genotype by environment interactions may exist (i.e., environment influences the expression of an animal's genes), which may complicate the statistical analyses.

Furthermore, accurate estimates of genetic merit require large and expensive progeny testing schemes. This is particularly true for low heritability traits where large progeny group sizes with recorded health phenotypes are necessary to obtain accurate estimates of genetic merit. New technologies provide an opportunity to reduce or alleviate some of these limitations.

## Usefulness of new 'omics' technologies in breeding for improved animal health

Advancements in 'omics' technologies and how the emerging data are interpreted and combined have the potential to overcome, or at least minimise, some of the aforementioned disadvantages of the traditional methods of genetic evaluation. The recent advancements in 'omics' technologies and their applications to animal breeding have been discussed in detail by Berry et al. [42]. Marker-assisted selection, combining the traditional genetic evaluation of an animal with its genotype at several loci, was originally proposed as a method of incorporating genomic information into genetic evaluations. However, few markers have been commercialised based on associations with quantitative traits related to animal health. The use of genetic markers for pre-screening animals for single gene genetic disorders or traits has, however, been successfully used in animal breeding [43].

**Figure 2:**
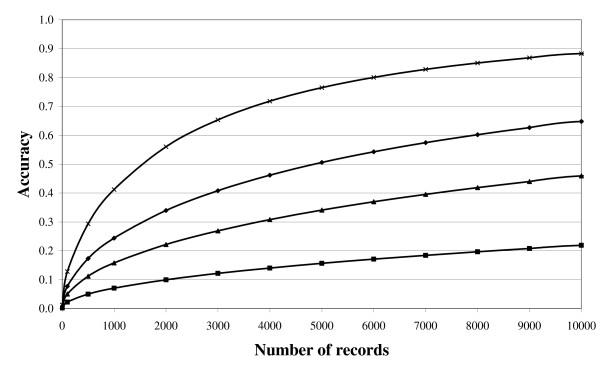
**Accuracy of genomic selection for a trait within a heritability of 0.03 (square), 0.15 (triangle), 0.35 (diamond) and 0.90 (x) across different numbers of animals genotyped and phenotyped (Daetwyler et al. **[47]).

Genomic selection [44] is now replacing the traditional methodology of genetic evaluation in dairy cattle, and is thought to be superior to marker-assisted selection. Genomic selection will also soon be implemented in beef cattle. Unlike marker-assisted selection which uses data on only tens of genetic markers, genomic selection is based on associating thousands of genetic markers, spread densely across the genomes of several thousand animals, with the range of phenotypes available on those animals. The end result is the estimate of the association between each marker and each phenotype. Miniaturised genotyping platforms with individual detection elements capable of simultaneously assaying up to 777,000 distinct SNP genetic markers, which are evenly distributed across the bovine genome are now commercially available (Illumina Inc., San Diego, CA). However, whole genome sequencing on large numbers of animals will soon become feasible [45], increasing the number of useful markers dramatically. Once each marker effect is known, then an animal with no available phenotype (e.g., a disease free newborn calf) can be genotyped and its direct genomic value (DGV) estimated based solely on its genotype. This is usually integrated with the traditional genetic evaluation of the animal to generate a genomic-estimated breeding value (GEBV). The advantage of such a method is that accurate estimates of genetic merit can be achieved exploiting knowledge of the genotype of the animal [46], even if the animal is very young. Reliability estimates of up to 50% have been reported in the Irish genomic selection-breeding programme for Holstein-Friesian dairy cattle in which no phenotypic data were available, which are higher than the approximate 30% reliability achieved previously using traditional methodology [47]. Nonetheless, large quantities of data are still required to accurately estimate the effect of each genetic marker (Figure 2, based on equations provided by Daetwyler et al. [48]).

## Concerns about breeding for improved resistance

Several concerns may be raised about the possible repercussions or limitations of breeding for improved animal health and resistance to disease, some of which have already been discussed in depth by Stear et al. [49]. Concerns include:

• sustainability of breeding for resistance in the face of continuous evolution of pathogens,

• the feasibility of selection for traits where little genetic variation may exist,

• impact of selection for resistance to a given disease on the genetic resistance to other diseases,

• the impact of selecting on a phenotype thought to reflect resistance to a disease, on the actual genetic predisposition to the disease (e.g., tuberculin test as a reflection of susceptibility to TB or somatic cell count as a reflection of resistance to mastitis),

• impact of selection for disease resistance on genetic gain in other traits affecting profitability, and

• the cost-benefit of developing a breeding programme to select for improved resistance to disease.

There is some concern that the rates of evolution of pathogen genomes are significantly more rapid that those of their vertebrate hosts [50]. In other words, pathogens will evolve faster than genetic gain in the host for resistance, thereby making any breeding programme unsustainable. Stear et al. [49] suggests that one potential mechanism to overcome such a concern would be to breed for improved resilience or tolerance to infection which would impose no selective pressure on the pathogen to evolve unlike breeding for resistance to the pathogen. However, there are certain conditions where improved tolerance of infection would not be beneficial in all circumstances. For example, improving tolerance to bovine tuberculosis would have little, if any, benefit in countries implementing eradication programmes for bovine tuberculosis where infected animals are culled.

An argument is sometimes used that progression from infection to disease is purely environmental or that the heritability of disease resistance is too low for breeding to be fruitful. However, this review clearly shows genetic variation in resistance to the majority of diseases in cattle. Therefore, if sufficient phenotypic data is available, then the impact of low heritability on genetic response is minimised. Furthermore, new technologies such as genomic selection, if exploited, will further reduce the impact of low heritability on genetic gain.

Interactions between diseases are expected [10,15] and, therefore, there is concern that selection for resistance to a given disease may increase susceptibility to other diseases. However, such a dilemma only materialises if antagonistic genetic correlations exist between traits. It is well known that sick animals are, on average, phenotypically more susceptible to infection with other diseases. This is substantiated by positive genetic correlations between many disease traits (discussed previously in this review). Nonetheless, accurate routine recording of disease incidence should be undertaken and monitored to ensure no unfavourable trends exist, as well as providing data to estimate genetic correlations with other health and disease traits, or more importantly expected response to selection based on the breeding goal. One option in an attempt to minimise any potential antagonistic effects could be to select for general immunity or tolerance to infectious diseases of major economic importance.

Due primarily to a lack of routinely available data for a given phenotype of interest, biological predictors are sometimes used as proxies. Such proxies include reaction to the tuberculin test as a predictor of susceptibility to tuberculosis, serological response to MAP as a predictor of susceptibility to MAP, and more commonly somatic cell count as a predictor of clinical mastitis. Although the correlations amongst most predictors and the traits being predicted are strong [9,17], they are not unity. This suggests that firstly additional genetic gain could be achievable by measuring the goal trait itself, but secondly selection on the proxy could have unfavourable repercussions for genetic susceptibility to the goal trait itself. For example, concern has been expressed that cows with low somatic cell count may be less able to respond to pathogenic challenges. Studies which experimentally induced intramammary infections reported that low somatic cell count was associated with increased risk or severity of infection [51,52]. However, studies investigating the relationship between somatic cell count and naturally occurring clinical mastitis failed to reveal any association between low somatic cell count and increased risk of clinical mastitis [53]. Nonetheless, the implications are not clear and should not be ignored when implementing breeding programmes for resistance using an indicator trait.

Response to selection for a given trait within a breeding goal is a function of, amongst others, the relative weight placed on that trait, the quantity of information available on that trait (which affects the accuracy of selection) and the phenotypic and genetic relationships with other traits within the breeding goal. The inclusion of a trait antagonistically correlated with performance within a breeding goal will reduce the genetic gain in the performance trait. Most studies, already discussed, have documented an antagonistic correlation between both animal health and resistance to disease and milk production (Table 1), implying that placing selection pressure on health and disease resistance will reduce genetic gain in milk production. However, selection on a balanced breeding goal will increase genetic gain in overall profitability if each of the traits is weighted appropriately by their respective economic values.

Routine access to accurate phenotypes is vital to achieving sustainable genetic gain in animal health and disease resistance. However, for rare diseases in particular, a cost-benefit analysis of generating sufficient data to estimate breeding values and then incorporate these values into a breeding programme should be evaluated relative to embarking on a project to control the disease through other means (e.g., vaccination). This is particularly true since expected responses to genetic selection will take a long time to develop although they are cumulative and permanent. Nonetheless, the development of 'omics' technology and associated analysis of the data may ameliorate the necessity for continuous routine access to such data. Furthermore, when evaluating the economic benefit of breeding for disease resistance, some account must be taken of the impact of selection on its effects on the transmission of disease through the population and, therefore, the challenge faced by all animals including those not selected for resistance [54].

## Future research

One of the biggest constraints of breeding for improved animal health or resistance to disease is routine access to accurate phenotypes (i.e., measurements) of health traits. Therefore, research must be undertaken on the development of inexpensive and humane methods of either challenging animals for disease resistance to generate phenotypes (e.g., the tuberculin test) or the development of accurate bio-markers that can be readily measured in large numbers of animals at a relatively low cost. Furthermore, increased collaboration between veterinarians and animal breeders on the definition and collection of the relevant phenotypes, as well as the most appropriate statistical model, based on biological soundness, is vital to achieving genetic gain.

Not alone do developments in animal breeding and the relevant 'omics' disciplines aid in breeding for increased resistance to infection, but elucidation of the genome of the host, coupled with the availability of the whole genome of the pathogen and how that interacts with the genome of the host, will help in determining the mode of infection and thus the development of effective vaccines or prophylactic treatments.

## Conclusions

There is overwhelming evidence that genetics make a significant contribution to the health and resistance to disease in cattle, and that the tools for simultaneous selection on these traits and other performance traits are available. Furthermore, because of differences in exposure rates as well as the lack of complete sensitivity and specificity of tests, heritability estimates for health and disease resistance traits discussed in this review are likely to be underestimates of the true heritability [55]. Additionally, new developments in 'omics' technologies provide a considerable resource that can be exploited to further increase genetic gain, especially in health and disease traits. Nonetheless, resistance to most diseases will be governed by a large number of genes, and mutations within genes. Therefore, absolute resistance is unlikely, and genetics alone is not the solution to improved animal health. Rather, it should be seen as an integral part of an overall programme for improved cattle health, both at farm level and national level.

## Competing interests

The authors declare that they have no competing interests.

## Authors' contributions

All authors contributed to the literature review and writing of the manuscript. All authors read and approved the final manuscript.
